# Evaluating Clinical Sequelae of the Carbapenem-Valproate Interaction: A Retrospective Analysis

**DOI:** 10.1093/ofid/ofae130

**Published:** 2024-03-08

**Authors:** Nick Petrucelli, Bryan D Hayes, Nidhi Shelat, Ramy H Elshaboury, Jeffrey C Pearson, Jennifer L Koehl

**Affiliations:** Department of Pharmacy, Massachusetts General Hospital, Boston, Massachusetts, USA; Department of Pharmacy, Massachusetts General Hospital, Boston, Massachusetts, USA; Department of Emergency Medicine, Division of Medical Toxicology, Harvard Medical School, Boston, Massachusetts, USA; Department of Pharmacy, Massachusetts General Hospital, Boston, Massachusetts, USA; Department of Pharmacy, Massachusetts General Hospital, Boston, Massachusetts, USA; Department of Pharmacy, Brigham and Women's Hospital, Boston, Massachusetts, USA; Department of Pharmacy, Massachusetts General Hospital, Boston, Massachusetts, USA

**Keywords:** carbapenem, interaction, seizure, valproate

## Abstract

**Background:**

Previous studies identified a rapid decrease in valproate serum concentrations when coadministered with a carbapenem; however, the specific consequences and subsequent therapy adjustments are not well described. We aimed to investigate the clinical and therapeutic implications of the carbapenem-valproate drug-drug interaction.

**Methods:**

This retrospective analysis included data from 2 large academic medical centers during January 2017 to June 2022. The primary outcome was incidence of seizures or behavioral events stratified by valproate indication. All adult patient encounters with concomitant administration of any carbapenem antimicrobial and valproate were included. Patients without prolonged exposure to valproate prior to hospitalization, without valproate levels pre– and post–carbapenem administration, with an admitting diagnosis of seizure, with exposure to other agents that decrease valproate concentrations, or who had a seizure during the hospitalization prior to carbapenem exposure were excluded.

**Results:**

Two hundred fifty-eight episodes of concomitant use among 78 unique adult patients were included. Valproate was used for seizure control in 41 patients (52.6%) and for mood-related disorders in 37 (47.4%). In those prescribed valproate for its antiepileptic properties, seizures occurred following carbapenem administration in 46.3% of encounters. In those taking valproate for mood-related disorders, 50.8% met the primary endpoint of behavioral disturbance.

**Conclusions:**

Our study demonstrates significant clinical implications of the carbapenem-valproate interaction. Clinicians should be aware of this interaction and consider alternative antimicrobial and/or antiepileptic agents whenever possible. Adding or increasing doses of antiepileptic agents and/or consultation with a neurologist prior to concomitant use should be considered when this combination cannot be avoided.

## Teaser

In this retrospective analysis, seizures and behavioral disturbances occurred following carbapenem administration in 46.3% and 50.8% of encounters, respectively, in patients with valproate exposure. Clinicians should be aware of this drug interaction and consider alternative antimicrobials when clinically appropriate.

Carbapenem antibiotic use is increasing globally secondary to rising antimicrobial resistance [1]. Carbapenems are frequently prescribed to hospitalized patients when first-line treatment options are unable to be employed due to antimicrobial resistance, previous treatment failure, severe β-lactam allergies, or other contraindications, thus limiting antimicrobial selection. Increasing use of carbapenems is particularly problematic in patients taking valproate due to a major drug-drug interaction characterized by significantly decreased serum concentrations of valproate, resulting in the potential for seizures or behavioral events.

The exact mechanism of the interaction is unknown, although several theories have been proposed [2, 3]. Valproate transporters and enzymes throughout the intestines and liver may be altered by carbapenems by eradicating enteric enzyme-producing bacteria. Alternatively, valproate demonstrates efflux from erythrocytes to the plasma via transporters that may be susceptible to carbapenem inhibition [2]. Regardless of the mechanism, administration of a carbapenem causes a rapid decline in valproate serum concentration commonly within the first 24 hours that may last for several weeks [3]. Although studies show a consistent decrease in valproate serum concentrations with the introduction of a carbapenem, the clinical consequences and subsequent therapeutic alterations have not been extensively described [2, 4–8]. Thus, we aim to investigate the clinical and therapeutic implications of this drug-drug interaction.

## METHODS

This retrospective analysis included hospitalized adult patients (≥18 years of age) at Massachusetts General Hospital and Brigham and Women's Hospital. Query of the electronic health records was used to identify patients who were concomitantly administered doses of any carbapenem antimicrobial and a valproate product within 24 hours of each other between January 2017 and June 2022 at the 2 institutions. Encounters with documented administration of other agents known to decrease valproate serum concentrations were excluded unless the patient had prolonged exposure to both medications prior to hospitalization (Supplementary Table 1). Patients without prolonged exposures to valproate prior to hospitalization and those without valproate levels drawn both before and after carbapenem administration within the same hospitalization were excluded from the study. Prolonged exposure to a prior-to-hospitalization medication was defined by at least 29 days of use as evidenced by dispense records available within the electronic health record. Encounters with an admitting diagnosis of seizure or a documented seizure during the hospitalization prior to carbapenem administration were also excluded from the valproate-for-seizure cohort.

Patients taking valproate for seizures were analyzed separately from those taking valproate for mood stabilization and every hospital visit was recorded as a distinct encounter. The primary outcome in the seizure control cohort was incidence of seizures (clinical or electrographic) as documented by a clinician in a patient progress note following carbapenem initiation during the same hospitalization. The primary outcome in the mood-related disorder cohort was behavioral-related events following carbapenem initiation during the same hospitalization, defined by the presence of at least 1 relevant keyword within a patient progress note (Supplementary Table 2) or clinician ordering of an inpatient psychiatric consult. Notes with identified keywords were evaluated to ensure documentation of an active behavioral issue in conjunction with the present hospitalization. Secondary outcomes included change in total valproate serum concentration after carbapenem administration, time to seizure or behavioral event following the first carbapenem dose, incidence of starting a new antiepileptic agent during the hospitalization, duration of valproate serum concentrations <50 μg/mL reflecting the lower limit of an acceptable serum concentration in patients with seizures, and incidence of valproate dose increases during the hospitalization [9]. Valproate concentrations were extracted from the window of hospital arrival to hospital discharge. The primary outcome was assessed among individual carbapenems comparatively. Secondary outcomes evaluating valproate serum concentrations and antiepileptic additions were limited to the seizure group.

### Data Collection and Analysis

All data points including baseline characteristics and outcome measures were recorded using Research Electronic Data Capture (REDCap) and data analysis was performed using SPSS software version 28 (IBM SPSS Statistics, IBM Corporation, Armonk, New York). Data collection was mostly performed by the primary author while all authors contributed to the adjudication process of difficult cases in which consensus among the group was pursued to ensure uniformity. Categorical variables were described as number (%), and continuous variables were described as mean (standard deviation) for normally distributed and median (interquartile range [IQR]) for nonnormally distributed data.

## RESULTS

**Figure 1.**
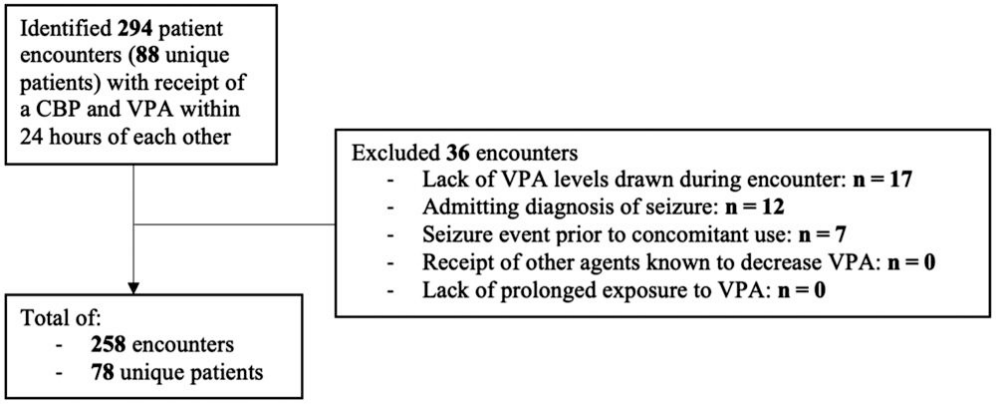
Study enrollment. Abbreviations: CBP, carbapenem; VPA, valproate.

In the 78 patients included in our analysis, there were 258 separate hospital encounters where valproate and a carbapenem were used concomitantly (Figure 1). Baseline characteristics are presented in Table 1. Valproate was used for seizure control in 41 patients (52.6%) and for mood-related disorders in 37 patients (47.4%). The most common admitting diagnosis was infectious diseases related (37.2% of encounters), with pneumonia being the most common indication for carbapenem use (24.8% of encounters) and meropenem being the most commonly administered carbapenem (Table 2). About 25% of encounters were characterized by an admitting diagnosis that was neurological in nature (ie, altered mental status, agitation, headache, stroke, transient ischemic attack, dementia, Parkinson's disease, or multiple sclerosis).

**Table 1.** Baseline Characteristics

Characteristic	N = 78 Patients
Age, y, median (IQR)	54 (40–75)
Weight, kg, median (IQR)	78.4 (67–91)
Male sex, No. (%)	44 (56.4)
VPA indication, No. (%)	
Seizure disorder	41 (52.6)
Mood-related disorder	37 (47.4)

Encounter characteristics	N = 258 encounters
Admitting diagnosis, No. (%)	
Infectious diseases	96 (37.2)
Neurological	66 (25.6)
Cardiovascular	61 (23.6)
Pulmonary	18 (7.0)
Oncologic	10 (3.9)
Gastrointestinal/genitourinary	7 (2.7)

Abbreviations: IQR, interquartile range; VPA, valproate.

**Table 2.** Carbapenem Use

Characteristic	N = 258 Encounters
Carbapenem, No. (%)	
Meropenem	126 (48.8)
Ertapenem	66 (25.6)
Imipenem-cilastatin	55 (21.3)
Imipenem-cilastatin-relebactam	6 (2.3)
Meropenem-vaborbactam	5 (1.9)
Median duration of CBP use, d (IQR)	6.2 (4.7–10.9)
CBP indication, No. (%)	
Pneumonia	64 (24.8)
Intra-abdominal infection	52 (20.2)
Urinary tract infection	45 (17.4)
Skin and soft tissue infection	38 (14.7)
Bloodstream infection	30 (11.6)
Bone or joint infection	24 (9.3)
CNS infection	5 (1.9)
Reason for CBP, No. (%)	
Active infection with MDRO	97 (37.6)
History of MDRO	73 (28.3)
Allergies	47 (18.2)
Unknown	41 (15.9)

Abbreviations: CBP, carbapenem; CNS, central nervous system; IQR, interquartile range; MDRO, multidrug-resistant organism.

**Figure 2.**
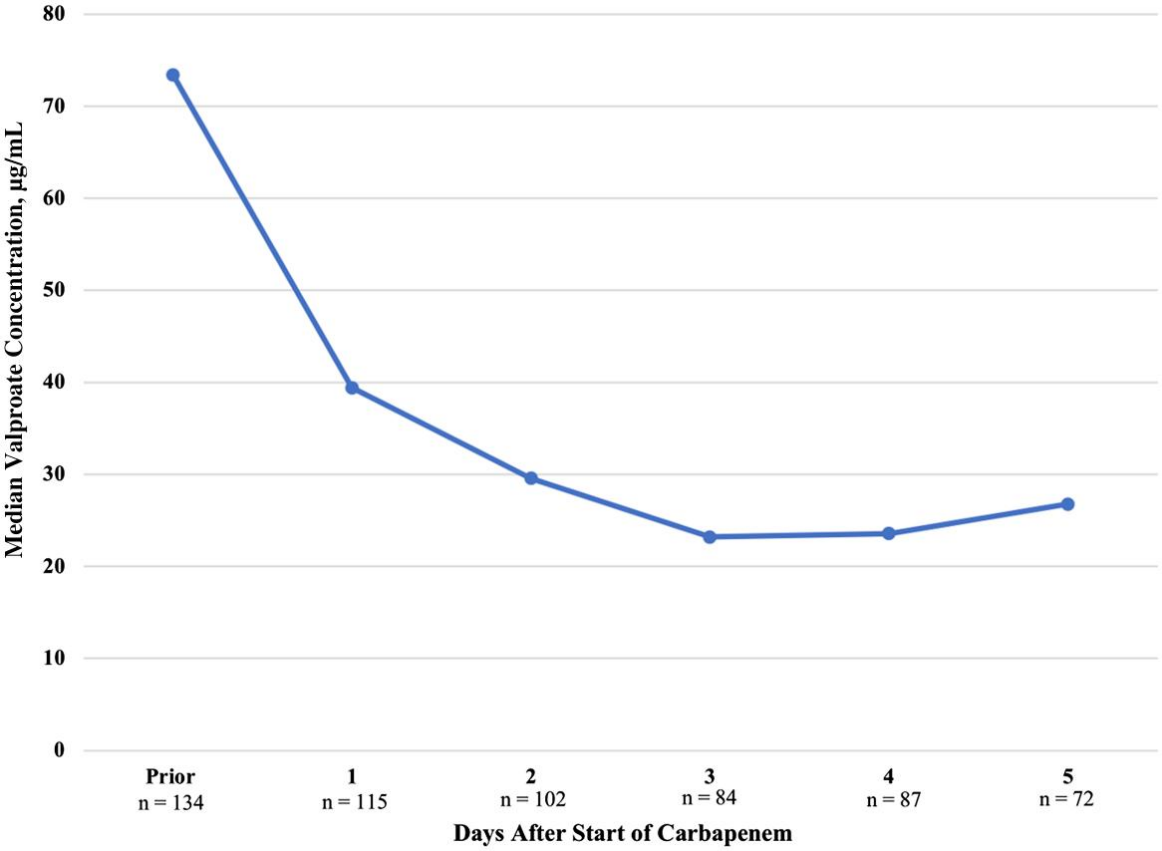
Change in valproate concentration in the valproate-for-seizure group (N = 134 encounters).

In the 134 encounters where valproate was used for seizure control, seizures occurred following carbapenem administration in 62 encounters (46.3%) representing 17 unique patients (41.5%). In those who experienced a seizure, 44 encounters (71%) had an associated valproate concentration <50 μg/mL documented within 24 hours of the event. The median time to seizure event was 2.6 days (IQR, 1.6–5.4 days). The median duration of serum valproate concentrations <50 μg/mL among those taking valproate for seizures was 8.9 days (IQR, 4.8–14.9 days) (Table 3). The median total percentage decrease in valproate concentration was 62%, and a median 46.3% decrease was noted within the first 24 hours of carbapenem initiation (Figure 2). In this cohort, 24% of encounters resulted in the initiation of a new antiepileptic agent (ie, levetiracetam, lacosamide, topiramate, phenytoin, carbamazepine, oxcarbazepine, or perampanel) following carbapenem use, while the dose of valproate was increased in 30.6% of encounters (Table 3). Additionally, 26.9% of encounters were noted to have at least 1 dose increase in an antiepileptic agent other than valproate following carbapenem use.

**Table 3.** Outcome Results

Outcome	No. (%)
VPA for seizure control	n = 134 encounters
Seizure	62 (46.3)
VPA level <50 μg/mL	44 (71.0)
Median time to event, d (IQR)	2.6 (1.6–5.4)
Median % decrease in VPA, μg/mL (IQR)	62 (54.9–82.1)
0–24 h	46.3 (38.4–54.8)
24–48 h	59.7 (47.6–65.7)
48–72 h	68.4 (62.3–75.6)
Median duration of VPA concentration <50 μg/mL, d (IQR)	8.9 (4.8–14.9)
Addition of new AED	32 (23.9)
VPA dose increase	41 (30.6)
Median % dose increase (IQR)	50 (35–75)
VPA for mood-related disorder	n = 124 encounters
Behavioral disturbance	63 (50.8)
Psychiatric consult ordered	38 (60.3)
Keyword identified	25 (39.7)
Median time to event, d (IQR)	5.2 (2.6–7.6)
VPA dose increase	40 (32.3)

Data are presented as No. (%) unless otherwise indicated.

Abbreviations: AED, antiepileptic drug; IQR, interquartile range; VPA, valproate.

In the 124 encounters where valproate was used for mood-related disorders, behavioral disturbances were identified in 63 cases (50.8%), of which 38 (60.3%) were ordered for a psychiatric consult and 25 (39.7%) were identified from a listed keyword. Within this group, the median time to event was 5.2 days (IQR, 2.6–7.6 days). The dose of valproate was increased in 32.3% of these encounters following carbapenem administration (Table 3).

The primary outcome of seizure or behavioral event occurred in 65 (51.6%), 27 (40.9%), and 33 (60%) encounters receiving meropenem, ertapenem, and imipenem-cilastatin, respectively.

## DISCUSSION

The effects of systemic carbapenem exposure on valproate serum concentrations are described in the literature [2–10]. We sought to corroborate this clinically significant drug-drug interaction by assessing how often seizures or behavioral changes are precipitated in the setting of decreased valproate serum concentrations. Roughly half of the included patient encounters had serious clinical consequences associated with this drug-drug interaction. A large majority of documented seizure events occurred in the context of subtherapeutic valproate serum concentrations leaving patients vulnerable to worsening underlying disease as early as day 1 of carbapenem therapy.

An observational study by Huang and colleagues assessed this drug-drug interaction and its clinical implications among 54 hospitalized patients [5]. Similar incidences of seizures (48%) and changes in serum valproate concentrations (73%) were reported, which occurred rapidly within the first or second day of carbapenem exposure. Additionally, Miranda Herrero and colleagues retrospectively reviewed 28 pediatric patients and similarly found that 54.5% of patients experienced seizures during coadministration [10]. Patients in this study had their valproate dose increased or were changed to a different antiepileptic as a result. Our study adds to the growing body of evidence that carbapenems pose serious risks when combined with valproate. The larger sample size we included allowed us to analyze the effects on patients taking valproate for mood stabilization in addition to seizure control, which has been an underrepresented population in previous publications.

Our study has several limitations. First, the patient screening protocol consisted of a 24-hour concomitant administration window in the electronic health record and therefore, any encounter with >24 hours between administration of a carbapenem and a valproate product may not have been captured. However, the sample size (number of patients and number of encounters) described in this analysis is adequate to infer clinical implications. The retrospective design of this study should be noted as well, considering the potential for biases and data incompleteness to impact the results presented. Identifying behavioral events as an outcome measurement is challenging and our approach utilizing keywords and consult services as a surrogate limits our ability to determine a true causal relationship. We additionally recognize the variability of valproate serum concentration obtainment and interpretation as we did not track when levels were drawn in relationship to the previous dose or concurrent albumin levels in the serum that are typically adjusted for in clinical practice. Finally, we did not monitor the incidence of seizure or behavioral event cessation in relationship to the mitigation strategies used to control for worsening of disease.

Overall, our study demonstrates notable clinical implications of the carbapenem-valproate drug-drug interaction within a robust sample size. Clinicians should be aware of this interaction and alternative antimicrobial agents should be considered whenever possible. We recognize that patient-specific factors such as true β-lactam allergies and multidrug-resistant pathogens may necessitate the use of a carbapenem. In these instances, adding or increasing doses of antiepileptic agents and/or consultation with a neurologist prior to concomitant use is advised to mitigate loss of seizure control or mood stability.

## CONCLUSIONS

In this retrospective analysis, seizures occurred following carbapenem antimicrobial use in 46.3% of patient encounters administered valproate for seizure control, with associated subtherapeutic valproate concentrations in most patients. Valproate concentrations decreased rapidly within the first 24 hours of carbapenem initiation, indicating that even 1 or 2 doses of a carbapenem can have consequences in patients taking valproate. About half of patients taking valproate for mood-related disorders experienced a behavioral disturbance following administration of a carbapenem. Future studies are warranted to determine the efficacy of the varying strategies utilized to avoid or overcome this drug-drug interaction.

## Supplementary Material

ofae130_Supplementary_Data

## Supplementary Data

Supplementary materials are available at *Open Forum Infectious Diseases* online. Consisting of data provided by the authors to benefit the reader, the posted materials are not copyedited and are the sole responsibility of the authors, so questions or comments should be addressed to the corresponding author.

## Notes

***Patient consent*.** The design of this work was approved by the local institutional committee, and a waiver of informed consent was granted as this study does not include factors necessitating patient consent (Massachusetts General Hospital, protocol number 2022P002184).
