# Supplementary material for: Evaluating Clinical Sequelae of the Carbapenem-Valproate Interaction: A Retrospective Analysis
Source: Open Forum Infect Dis. 2024 Mar 8;11(3):ofae130. doi: 10.1093/ofid/ofae130 (PMC10960597; doi:10.1093/ofid/ofae130)
Supplement: ofae130_Supplementary_Data [file ofae130_supplementary_data.docx]

**Tables: 2**

- **Supplemental Table 1:** Medications Searched that Reduce Valproate Serum Concentrations
- **Supplemental Table 2**: Keywords Searched to Identify Behavioral Events

**Supplemental Table 1: Medications Searched that Reduce Valproate Serum Concentrations^1^**

| carbamazepine  cholestyramine  estrogen derivatives  ethosuximide  methotrexate  perampanel  phenytoin/fosphenytoin  primidone  protease inhibitors  rifampin  topiramate |
| --- |

**Supplemental Table 2. Keywords Searched to Identify Behavioral Events**

| Agitat^A^  Anxiety  Anxious  Biting  Breaking or pulling out line  Climbing out of bed  Climbing over the side rails  Combative  Delusion^A^  Disrobing  Disruptive  Hallucin^A^  Hitting  Inappropriate  Kicking  Not cooperative  Paranoid ideation  Pinching  Pulling out tubes  Punching  Resist^A^  Screaming  Shouting  Spitting  Swearing  Uncooperative  Violence  Yelling |
| --- |

A: Allowed for any possible suffix of the word

**References:**

1. Abbott Pharmaceuticals. Depakote (divalproex)[package insert]. U.S. Food and Drug Administration website. Accessed January 20, 2024.
